# Inhibition of airway remodeling and inflammatory response by Icariin in asthma

**DOI:** 10.1186/s12906-019-2743-x

**Published:** 2019-11-19

**Authors:** Lingli Hu, Lulu Li, Hongying Zhang, Qiuping Li, Shan Jiang, Jian Qiu, Jing Sun, Jingcheng Dong

**Affiliations:** 1 Department of Integrative Medicine, Huashan Hospital, Fudan University, Shanghai, China; 2 Institute of Integrated Traditional Chinese and Western Medicine, Huashan Hospital, Fudan University, Shanghai, China

**Keywords:** Airway remodeling, Icariin, Asthma, Proliferation, MAPK/Erk pathway

## Abstract

**Background:**

Icariin (ICA) is the major active ingredient extracted from Chinese herbal medicine Epimedium, which has the effects of improving cardiovascular function, inducing tumor cell differentiation and increasing bone formation. It is still rarely reported that ICA can exert its therapeutic potential in asthma via anti-airway remodeling. The point of the study was to estimate the role of ICA in anti-.

airway remodeling and its possible mechanism of action in a mouse ovalbumin.

(OVA)-induced asthma model.

**Methods:**

Hematoxylin and Eosin Staining were performed for measuring airway remodeling related indicators. ELISA, Western blot and Immunohistochemistr-.

y (IHC) were used for analyzing the level of protein. RT-PCR was used for analyzing the level of mRNA.

**Results:**

On days 1 and 8, mice were sensitized to OVA by intraperitoneal injection. From day 16 to day 43, previously sensitized mice were exposed to OVA once daily by nebulizer. Interventions were performed orally with ICA (ICA low, medium and high dose groups) or dexamethasone 1 h prior to each OVA exposure. ICA improves pulmonary function, attenuates pulmonary inflammation and airway remodeling in mice exposed to OVA. Histological and Western blot analysis of the lungs show that ICA suppressed transforming growth factor beta 1 and vascular endothelial growth factor expression. Increase in interleukin 13 and endothelin-1 in serum and bronchoalveolar lavage fluid in OVA-induced asthmatic mice are also decreased by ICA. ICA attenuates airway smooth muscle cell proliferation, as well as key factors in the MAPK/Erk pathway.

**Conclusions:**

The fact that ICA can alleviate OVA-induced asthma at least partly through inhibition of ASMC proliferation via MAPK/Erk pathway provides a solid theoretical basis for ICA as a replacement therapy for asthma. These data reveal the underlying reasons of the use of ICA-rich herbs in Traditional Chinese Medicine to achieve good results in treating asthma.

## Background

As one of the common ailments and frequently-occurring diseases, asthma severely reduces the quality of life and affects human health. However, the mechanism of occurrence and development of asthma have not yet been accurately clarified. Numerous research have suggested in addition to inflammation and airway hyperresponsiveness (AHR), pathological changes in the bronchial airway structure known as airway remodeling have occurred [1]. The major components of airway remodeling include four aspects: 1) fibrosis with deposition of abnormal extracellular matrix components in the basement membrane layer beneath the epithelium; 2) goblet cell hyperplasia and increased mucus secretion; 3) angiogenesis; 4) increased thickness of smooth muscle due to muscle cell hyperplasia [2]. For airway remodeling has become a characteristic feature of asthma, seeking effective controlling medications for airway remodeling and clarifying the molecular mechanism are of great importance [3].

As one of the main factors regulating airway remodeling, transforming growth factor beta 1 (TGF-β1) could promote fibroblast precursors differentiate into myofibroblasts and the proliferation of myofibroblasts [4]. TGF-β1 could also accumulate extracellular matrix by activating the Smad protein family. In addition, it has been shown that TGF-β1 contributes to the migration of airway smooth muscle cell (ASMC) to the epithelial layer, a mechanism that promotes tissue remodeling [5]. Vascular endothelial growth factor (VEGF), a multifunctional angiogenic regulator [6], is overexpressed in asthmatics, and its expression level is positively correlated with disease activity and negatively correlated with the bronchial diameter and AHR [7, 8]. Relevant experimental evidence indicates that VEGF induces structural remodeling and inflammation in lung tissue of VEGF transgenic mice [9]. VEGF is able to induce angiogenesis in the murine airway and lung. All these properties of VEGF and TGF-β1 suggest that they are classical markers in airway remodeling.

When studying the pathogenesis of asthma, many concerns have been focused on ASMC as this type of cell is a crucial effector of AHR [10–13]. Apart from its contractile function, ASMC proliferation is another indispensable aspect of airway remodeling [14]. Numerous studies are devoted to the exploration and identification of cytokines, chemokines, growth factors and even signaling pathways involved in airway remodeling. Interleukin 13 (IL-13), epidermal growth factor and endothelins have been shown to take part in the occurrence and development of the remodeling process [15]. The ability of these cytokines to promote the formation of airway remodeling by accelerating cell proliferation alone or participating in other activities such as inflammation or mucus production has attracted the attention of researchers. When it comes to signaling pathways involved in remodeling, recent studies have indicated that blocking the mitogen-activated protein kinase (MAPK) signaling pathway, a pathway closely related to a variety of cellular functions including cell proliferation, could inhibit ASMC proliferation and thus relief remodeling [16–18].

Epimedium, a Chinese medicinal herb, exhibited anti-inflammatory effects in many conditions, such as lipopolysaccharide-mediated inflammation in macrophages, HeLa cell line and murine chondrocytes, high glucose-induced inflammation in human umbilical venous endothelial cells [19–22]. Also, Epimedium harbors the qualities of anti-oxidant and anti-allergic [23–26], which are a benefit to protect lung tissue and relieve asthma. Epimedium and its flavonoid components have been proven to exert anti-oxidative effects on cardiocytes and vein endothelial cell oxidative injury induced by H_2_O_2_, and are effective against oxidative-stress related acute myocardial anoxia and Alzheimer’s disease in animal rodent models and in vitro studies [27–32]. Icariin (ICA), the main active ingredient of Epimedium, performs multiple functions, such as improving cardiovascular function, inducing tumor cell differentiation and increasing bone formation. These anti-inflammatory, anti-oxidant and anti-allergic properties of Epimedium are hypothesized to protect lung tissue and relieve asthma. ICA is isolated from Epimedium and is reported to have a vast range of functions, such as anti-atherosclerosis, cardiovascular function improvement and antitumor [33], preventing platelet activation, blocking ASMC proliferation and migration, rescuing endothelial dysfunction, restoring DNA damage and inhibiting inflammatory response may be mechanisms to achieve these effects [34–36]. Although these features of ICA are somewhat associated with anti-airway remodeling, few studies have demonstrated the efficacy of ICA in the experimental model of asthma, not to mention the possibility of resistance to remodeling.

To investigate the potential of ICA for preventing asthma and airway remodeling, we determined the changes in pulmonary functions of ovalbumin (OVA)-induced asthmatic mice, and examined the modifications of airway remodeling correlated markers. To explore the functional mechanism of ICA treatment for airway remodeling, proliferation associated markers IL-13 and endothelin-1(ET-1), and activity of MAPK/Erk signaling pathway were detected, focusing on effects of ICA on cell proliferation in the pathogenesis of airway remodeling. The current study is crucial to validate and strengthen the phytotherapeutic use of ICA-rich herbs in asthma and airway remodeling related diseases.

## Methods

### Chemicals and reagents

OVA (grade V) (Sigma, Taufkirchen, Germany), ICA (Mansite Biotechnology Co.Ltd., Chengdu, China), dexamethasone (DXM) (Xinhua Pharmaceutical Co., Ltd., Shandong, China), RIPA Lysis Buffer (Beyotime, Shanghai, China), BCA Protein Assay Kit (Beyotime, Shanghai, China), antibodies against TGF-β1 and VEGF (Santa Cruz Biotechnology, CA, USA), antibody against GAPDH (Cell Signaling, Houston, TX, USA), HRP-labeled secondary antibody (Santa Cruz Biotechnology, CA, USA), mouse ELISA kit of IL-13 and ET-1 (ExCell Bio, Taicang, China), TRIZOL used to extract total RNA (Takara Bio, Kusatsu, Shiga, Japan), iScriptcDNA Synthesis Kit used to synthesize cDNA (Bio-Rad, Hercules, CA, USA).

### Animals

Mouse is the most common species studied in animal models of asthma. OVA induced asthma model offers many opportunities for deepening understanding of the pathogenesis of this disease. In this study, OVA-challenged mice were used as experimental models of asthma. Female BALB/c mice (5 ± 1 weeks old, 18–22 g) were purchased from the Department of Laboratory Animal Science, School of Medicine, Fudan University and housed in an environment where there are no specific pathogen, temperature and humidity are controlled, and water and food are given ad libitum access to. The whole protocol was approved by the Institutional Animal Care and Use Committee of Fudan University (SYXK (hu) 2010–0099). All sections of this research adhere to the ARRIVE Guidelines for reporting animal research. A completed ARRIVE Guidelines checklist is included in Additional file 1.

### Treatment of mice

**Fig. 1:**
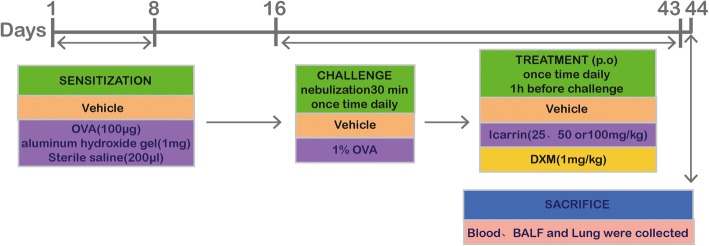
Schematic representation of the protocol for ovalbumin (OVA)-induced airway remodeling in mice, and treatment with icariin (ICA) or dexamethasone (DXM)

For the induction of airway remodeling in mice, the protocol described in Fig. 1 was employed. Mice were randomly divided into 6 groups of 10 mice each.

On days 1 and 8:

Mice were intraperitoneally injected with 100 μg OVA and 1 mg aluminum hydroxide gel in 0.2 ml sterile saline solution.

On days 16–43:

Control group: mice were received the same volume of vehicle.

OVA group: mice were inhaled 1% OVA once a day for 0.5 h to duplicate asthmatic remodeling model.

ICA group: mice were treated by gavage 0.3 ml ICA solution 1 h before OVA challenge. ICA 25, 50, and 100 mg/kg were low, medium, and high doses respectively.

DXM group: mice were treated by gavage 1 mg/kg DXM 1 h before OVA challenge.

Mice were euthanized by cervical dislocation after lung function test on day 44, then the BALF and blood (serum) were collected for cytokines determination, and lung tissues were collected for histopathological analysis and proteins detection.

### Cell culture and treatment

ASMC were incubated with vehicle, DXM 100 μM or ICA (5, 10, or 100 μM) for 24 h to study the effect of ICA on cells.

### Pulmonary function test

For the measurement of pulmonary function of asthmatic mice, the protocol described by Pichavant et al. [37] was employed. Following the respiratory function measurements, mice lungs were collected for analysis.

### Analysis of BALF

The right lung of the mouse under anesthesia was ligated. The left lung was lavaged three times with tracheal cannula with 0.2 ml PBS, and the recovery rate was approximately 80%. Immediately centrifuged lavage supernatant was collected and stored at − 80 °C for ELISA measurements.

### Histopathological assessment

Mice lungs sections (4 μm) were stained with hematoxylin and eosin (H&E), then histopathological assessment in the bronchus was performed by a light microscope at high power (200×) in a blind fashion. The specific method was as follows: randomly choose 3 bronchus per slide and take photos. The selection criteria were that the bronchus were circular with the diameter about 200–400 μm and major/minor axis ≤ 1.2. Then, the following indicators including bronchial basement membrane perimeter (Pbm), smooth muscle area (Wam), inner airway area (Wai), amount of ASMC nucleus (N) should be measured by Image-pro Plus medical image analysis system. All the data measured was standardized using Pbm.

### Immunohistochemistry (IHC)

IHC was performed using a two-step EnVision/HRP technique according to the manufacturer’s instruction. The expression of TGF-β1 (anti-TGF-β1, 1:100 dilution) and VEGF (anti-VEGF, 1:100 dilution) were quantitatively evaluated by using Olympus Cx31 microscope with Image-pro Plus medical image analysis system. The positive area and optical density (OD) of TGF-β1 and VEGF positive cells were determined by measuring three randomly selected microscopic fields (25 × 10) per slide. IHC index was defined as an average integral optical density (AIOD) (AIOD = positive area×OD/total area).

### Western blot

Protein extraction, concentration determination and the following process were consistent with our previous experiment [38]. The dilution ratio of antibodies used in this research were as follows: anti-TGF-β1 (1:1000 dilution), anti-VEGF (1:1000 dilution), anti-GAPDH (1:5000 dilution) and HRP-labeled secondary antibody (1:5000 dilution).

### ELISA analysis of IL-13 and ET-1

The protein expressions of IL-13 and ET-1 in BALF and serum were detected by corresponding antibody ELISA Kit according to the manuals.

### Analysis of mRNA expression

Quantitative real-time polymerase chain reaction was used to detected mRNA expression and the process was consistent with our previous expriments [38]. The primers used in this experiment were as follows: Rat Erk1/2: CCACTTTACCACAACACGCTAGA (forward), GATGAAGAGGCCTCCAATGACT (reverse). Rat p21 ras.ATCGAGACCTCGGCCAAGA(forward),TCACGCACCAACGTGTAGAAG(reverse). Rat GAPDH: TCCTGCACCACCAACTGCTTAG (forward), AGTGGCAGTGATGGCATGGACT (reverse).

### Statistical analysis

Data were presented as mean ± SEM. An unpaired Student’s t-test and an ANOVA analysis with a Bonferroni post hoc test were used for single and multiple comparisons between two or more groups, respectively. The *P* < 0.05 was considered statistically significant.

## Results

### ICA reduced OVA-induced airway remodeling

**Fig. 2:**
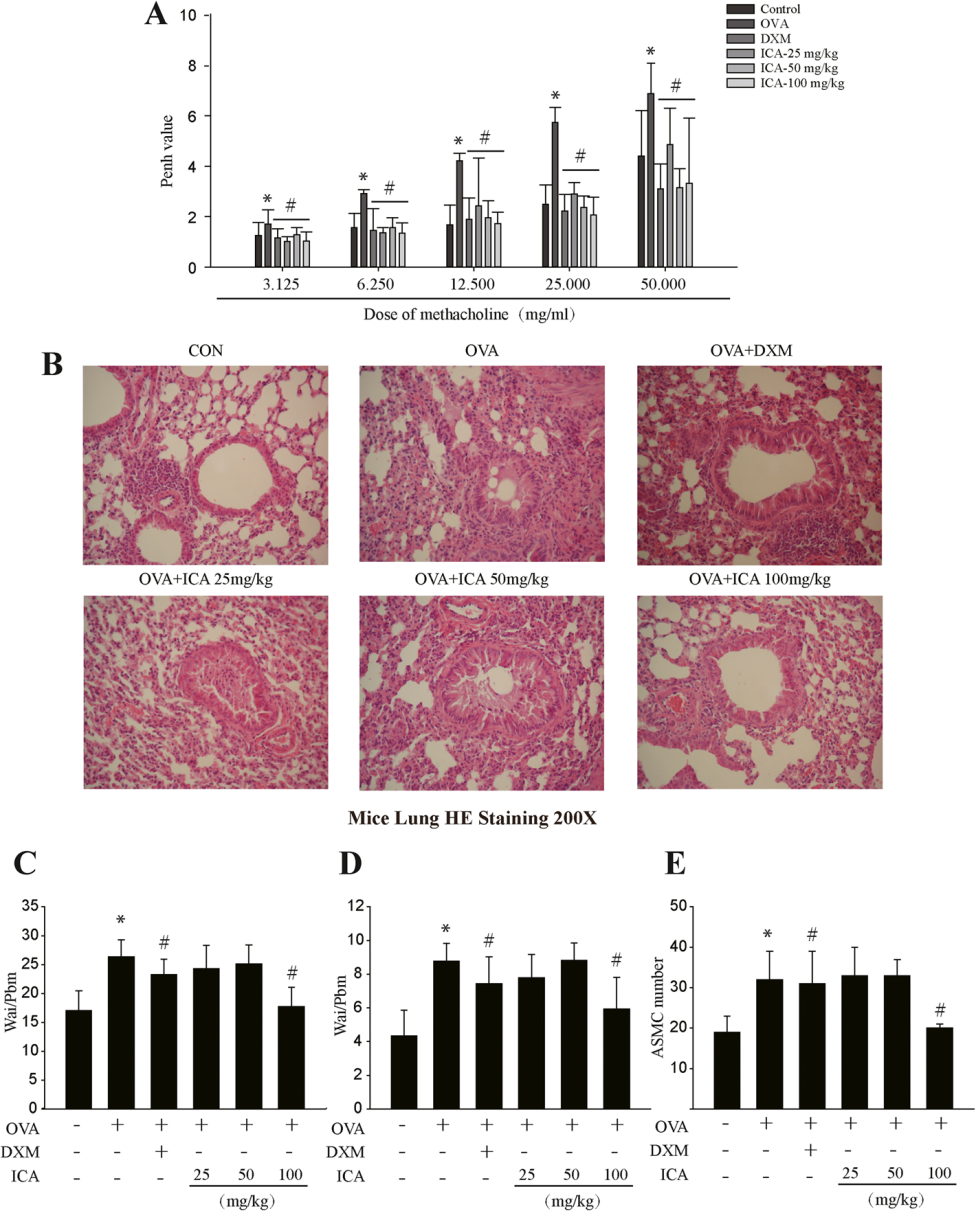
Changes in pulmonary function, pulmonary inflammation and airway remodeling following OVA exposure in mice treated with ICA. Wild-type BALB/c female mice were received vehicle or 0.2 ml sensitizing mixture (100 μg OVA and 1 mg aluminum hydroxide gel in sterile saline solution) on days 1 and 8. Then, mice were inhaled 1% OVA once a day for 0.5 h on days 16–43, and mice received vehicle or ICA (25, 50, or 100 mg/kg) or 1 mg/kg DXM 1 h before OVA inhalation. **a** Responsiveness to inhaled methacholine was measured using whole-body barometric plethysmography in awake, unrestrained mice. **b** H&E staining was performed in mice lungs sections to observe the inflammation degree and measure the remodeling related indicators: Wam/ Pbm (**c**)、Wai/ Pbm (**d**)、N (**e**). Data are shown as mean ± SEM (*n* = 10). *P < 0.05 versus control group and ^#^P < 0.05 versus OVA group

The effect of ICA on OVA-induced airway remodeling was measured by testing pulmonary function and performing H&E staining in the lung sections from OVA sensitized and ICA or DXM treatment mice. Penh (enhanced pause), an index of bronchoconstriction, was measured at baseline and after sequential delivery of increasing concentrations of methacholine (3.125–50 mg/ml). As shown in Fig. 2a, OVA-induced upregulation of Penh levels was reduced by ICA in a dose-dependent manner, and its therapeutic effect was similar to or even better than the treatment of DXM, currently the most effective medicine for asthma in the clinic. The characteristic manifestations of asthma are consist of a marked increase in airway inflammatory cell infiltration and the appearance of airway remodeling. Current study has shown that lung tissues of mice in OVA group were filled with inflammatory cells, and this typical feature was significantly reduced by ICA in a dose-dependent manner (Fig. 2b). In addition, airway remodeling indicators of Wam/Pbm, Wai/ Pbm, and N were tested by Image-pro Plus software, all these parameters were markedly increased in OVA-induced model group, while these elevated indicators were alleviated by ICA or DXM in the pretreatment groups (Fig. 2c-e).

### ICA attenuated the expression of airway remodeling related factors

**Fig. 3:**
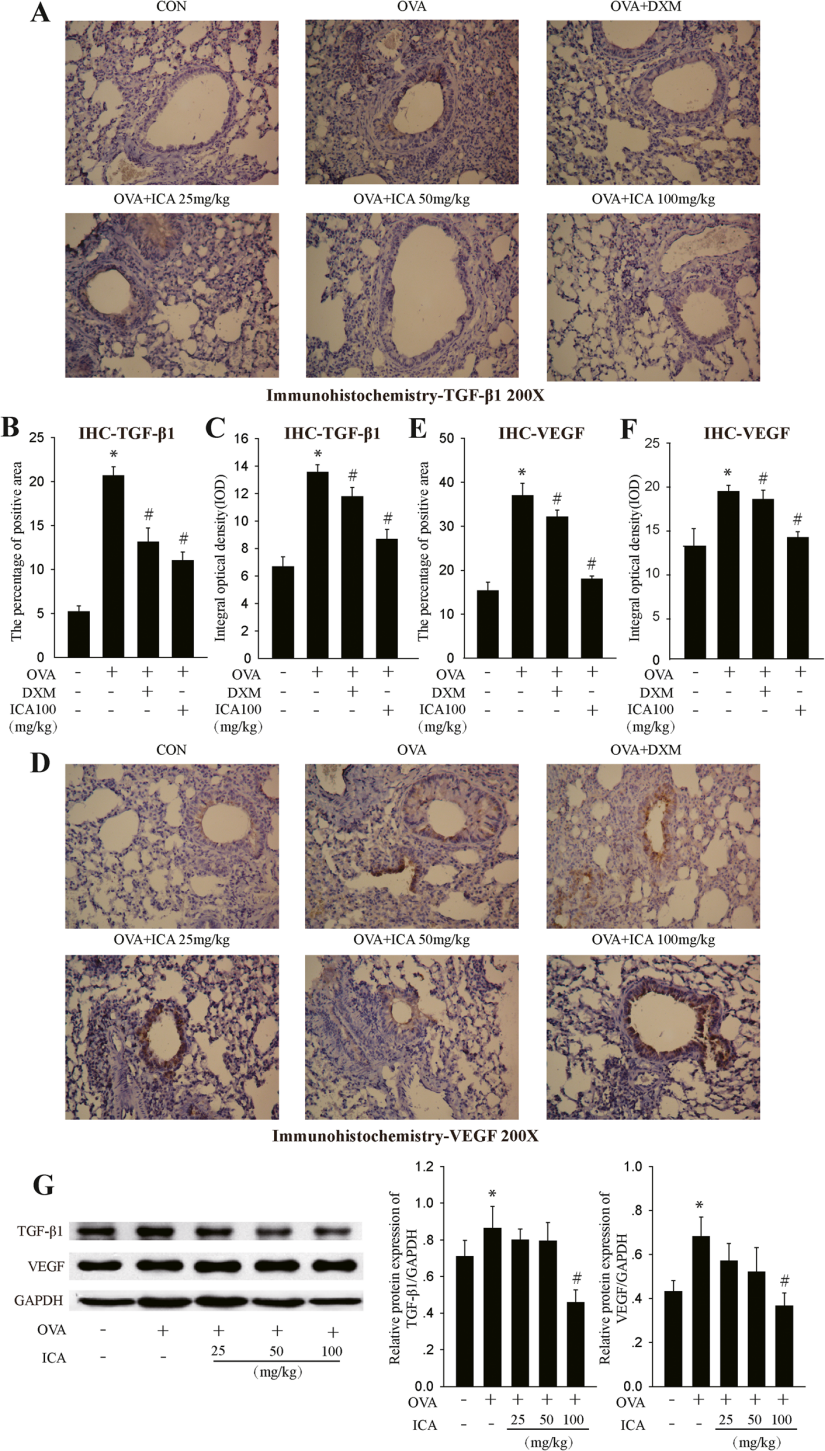
Influence of ICA on expression of remodeling markers. Wild-type BALB/c female mice received vehicle or 0.2 ml sensitizing mixture (100 μg OVA and 1 mg aluminum hydroxide gel in sterile saline solution) on days 1 and 8. Then, mice were inhaled 1% OVA once a day for 0.5 h on days 16–43, and mice received vehicle or ICA (25, 50, or 100 mg/kg) or 1 mg/kg DXM 1 h before OVA inhalation. **a** TGF-β1 IHC in lung sections. **b** The percentage of TGF-β1 positive area . **c** The OD of TGF-β1 positive cells. **d** VEGF IHC in lung sections. **e** The percentage of VEGF positive area . **f** The OD of VEGF positive cells. **g** Western blot analysis of TGF-β1 and VEGF expression in mice lungs tissues. Results are shown as mean ± SEM (*n* = 10) . ^*^P < 0.05 versus control group and ^#^P < 0.05 versus OVA group

As ICA had potently alleviated the histopathological phenotype of airway remodeling, we further tested whether ICA had an effect on the expression of airway remodeling related factors. IHC in lung sections indicated that in each group, there was universal cytoplasmic TGF-β1 positive immunostaining and scatter nuclear TGF-β1 positive immunostaining. Analytic data showed ICA reversed OVA-induced TGF-β1 over-.

expression (Fig. 3a), including the percentage of TGF-β1 positive area (Fig. 3b) and the OD of TGF-β1 positive cells (Fig. 3c). VEGF, another important mediator of the neoangiogenesis component of remodeling in asthma, its IHC results in lung sections were similar to that of TGF-β1 (Fig. 3d-f). Meanwhile, the data of western blot performed to assess the protein expression of TGF-β1 and VEGF were consistent with the observation of IHC (Fig. 3g).

### ICA reduced the secretion of factors that promote airway remodeling

**Fig. 4:**
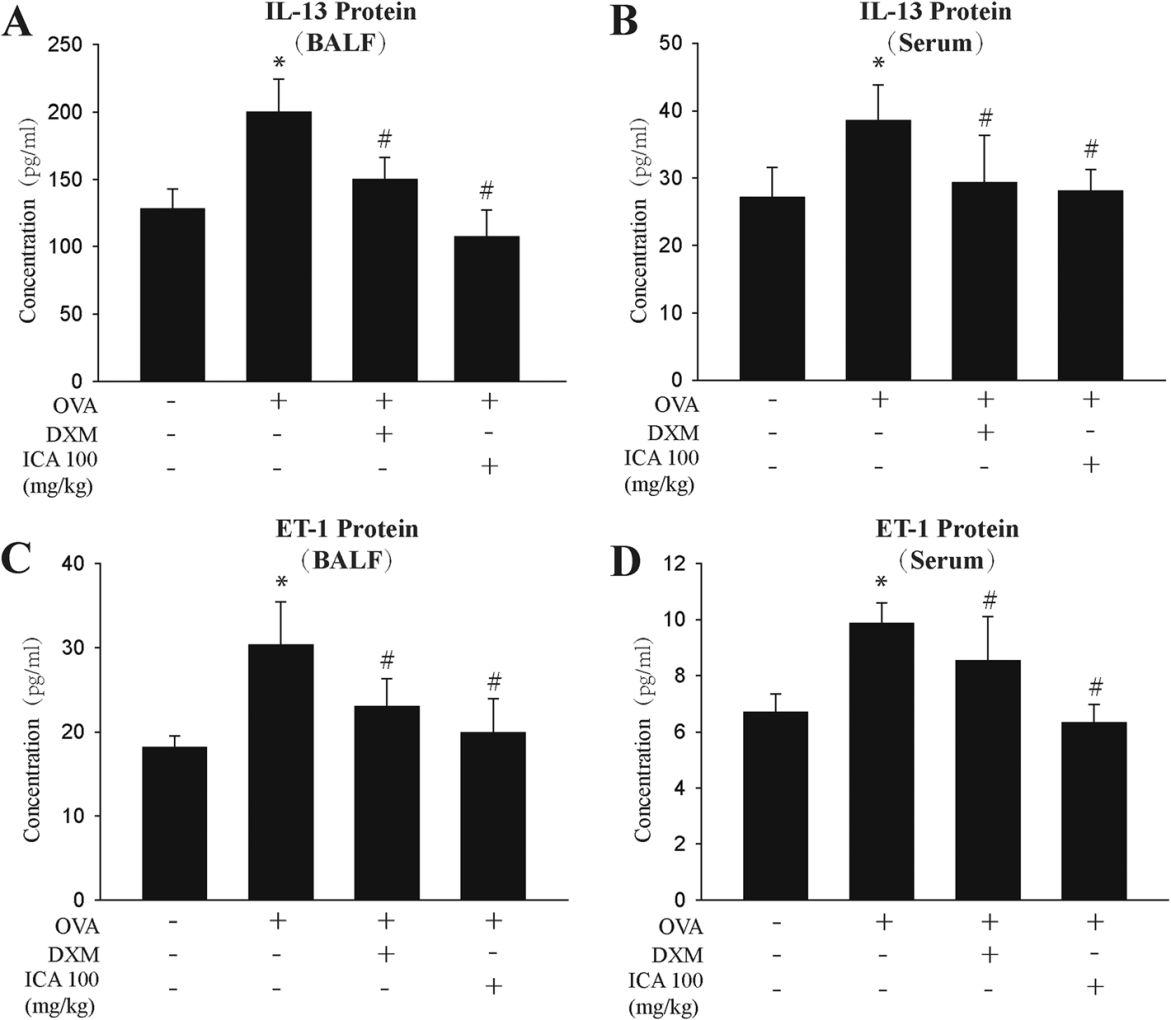
ICA inhibited the release of IL-13 and ET-1 in BALF and blood serum. Wild-type BALB/c female mice received vehicle or 0.2 ml sensitizing mixture (100 μg OVA and 1 mg aluminum hydroxide gel in sterile saline solution) on days 1 and 8. Then, mice were inhaled 1% OVA once a day for 0.5 h on days 16–43, and mice received vehicle or ICA (25, 50, or 100 mg/kg) or 1 mg/kg DXM 1 h before OVA inhalation. **a, b** ELISA measurement of IL-13 in BALF and in blood serum. **c, d** ELISA measurement of ET-1 in BALF and in blood serum. Data are shown as mean ± SEM (*n* = 10) . ^*^P < 0.05 versus control group and ^#^*P* < 0.05 versus OVA group

Considering the previous data showing ICA acts a pivotal part in airway remodeling mitigation, determining its functional mechanism is the next step we will take. A number of investigators have reported that IL-13 plays a key role in the induction of airway remodeling in airway epithelium, smooth muscle, fibroblasts, and endothelium [39, 40]. In order to determine whether ICA attenuates airway remodeling by affecting IL-13 secretion, the protein expressions of IL-13 were tested via ELISA. The results indicated that ICA significantly inhibited the release of IL-13 from BALF and serum (Fig. 4a, b).

As a potent bronchoconstrictor and endogenous vasoconstrictor peptide with growth-promoting properties [41–43], ET-1 is involved in vascular and airway hyperresponsiveness caused by cigarette smoke [44]. ET-1 has also been shown to affect airway remodeling and hyper-reactivity in a murine asthma model [45]. Confirmation of the role of ICA on ET-1 expression by detecting protein expression of ET-1 in BALF and serum via ELISA. The data revealed that OVA-induced upregulation of ET-1 protein level was down-regulated in the presence of ICA (Fig. 4c, d).

The above results may suggest that ICA can inhibit the release of cytokines that promote airway remodeling.

### ICA inhibits the proliferation of ASMC and the activity of proliferation-related signaling pathway

Since previous data showed that ICA can inhibit the release of cytokines that promote airway remodeling. Furthermore, both IL-13 and ET-1 have the property of promoting cell proliferation. ET-1 could promote ASMC division, induce ASMC proliferation, and inhibit ASMC apoptosis [46]. IL-13 has been shown to activate the pathway of JAK/STAT that cause the proliferation of human smooth muscle cells [47]. To study whether ICA has an effect on ASMC proliferation, cells were incubated with increasing concentration of ICA or DXM for 24 h, and the effects of ICA and DXM were examined by flow cytometry. As shown in (Table 1), ICA increased the proportion of ASMC in G0/G1 phase and decreased the proportion of cells in S + G2/M phase in a dose-dependent manner. The above data indicated that ICA could inhibit the proliferation of ASMC.

**Table 1:** The populations of ASMC in each phase of the cell cycle.ASMC were incubated with vehicle, DXM 100 μM or ICA (5, 10, or 100 μM) for 24 h

Groups	G0/G1(%)	S+G2/M(%)
Vehicle	61.2±1.47	38.4±1.86
DXM 100μM	86±1.65 *	23.2±1.74 *
ICA 5μM	60.5±2.08	39.5±2.37
ICA 10μM	62.8±2.57	36.2±1.94
ICA 100μM	72.9±3.04 *	27.2±2.29*

The populations of ASMC in each phase of the cell cycle were detected by flow cytometry using PI staining. Data are shown as mean ± SEM, ^*^P < 0.05 versus control group (*n* = 6)

**Fig. 5:**
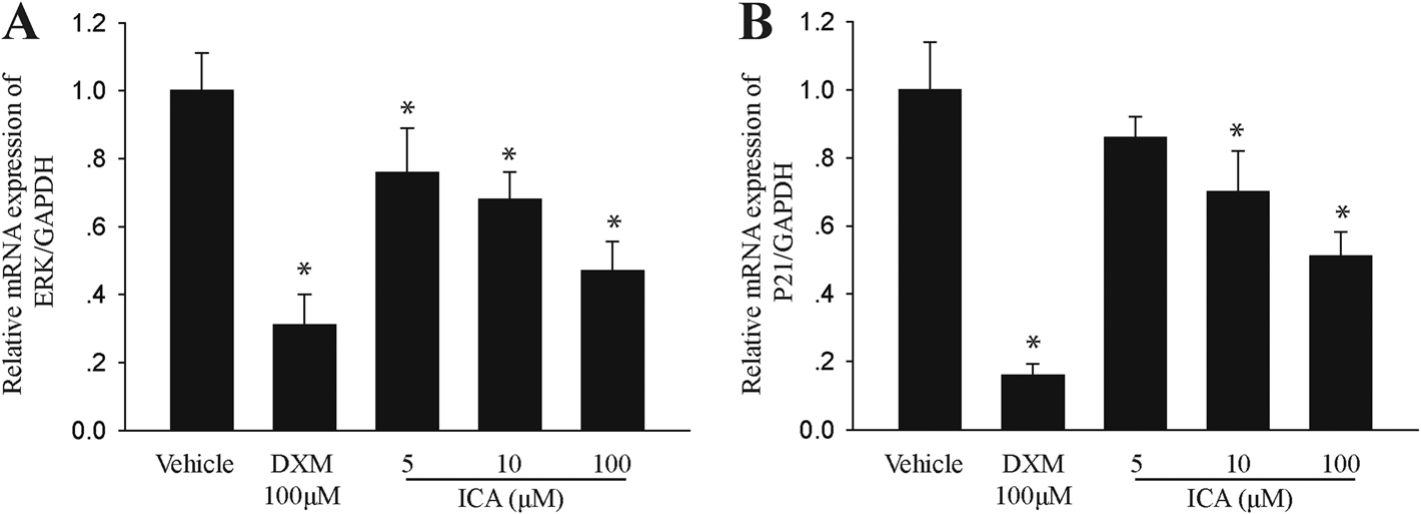
ICA inhibits the activity of proliferation-related signaling pathway. ASMC were incubated with vehicle, DXM 100 μM or ICA (5, 10, or 100 μM) for 24 h. **a**, **b** Expression of Erk1/2 and p21ras were tested via quantitative real-time polymerase chain reaction. Data are shown as mean ± SEM, ^*^P < 0.05 versus control group (*n* = 6)

The effects of ICA on the expression of key cytokines Erk1/2 and p21ras on the MAPK/Erk signaling pathway were further identified to explore the in-depth mechanism of ICA inhibition of cell proliferation. The results indicated that ICA decreased mRNA levels of Erk1/2 and p21ras in a dose-dependent manner (Fig. 5a, b). Therefore, targeting the MAPK/Erk signaling pathway might be the deep mechanism for ICA to ameliorate airway remodeling.

## Discussion

This research was to investigate the role of ICA in OVA- mediated airway remodeling as well as to explain its functional mechanism. OVA exposure induced the formation of airway remodeling in mice and upregulated the expression of markers associated with airway remodeling. Apart from ameliorating airway remodeling, ICA also has the ability to reduce OVA-induced ASMC proliferation. Mechanistically, ICA could dose-dependently abrogate OVA-induced the overexpression of IL-13, ET-1,and mRNA levels of Erk1/2 and p21ras in MAPK/Erk signaling pathway, which are involved in the OVA-induced cell proliferation .

Asthma is a chronic disease related to AHR and airway remodeling. Airway structural and functional changes due to repeated and persistent damage are defined as pathological airway remodeling [48]. Moreover, persistent and progressive damage to asthmatic lung function is related to airway remodeling [49]. The common and classic strategy for bronchial asthma treatment is to rapidly relieve acute asthma symptoms, reduce bronchoconstriction and airway inflammation by inhaling long-acting β2-adrenergic agonists and inhaled corticosteroids [50]. However, some patients still do not respond to this effective combination therapy or have low compliance or adverse side effects due to long-term continuous use [51, 52].Therefore, seeking for complementary therapy, especially phytotherapy, and effective controlling medications for airway remodeling has become the new direction of asthma research [53].

In the present study, we found that pretreatment of ICA could improve lung function, relieve airway inflammation and airway remodeling in asthmatic mice model. In addition, ICA synchronously decreases the expression of VEGF and TGF-β1, the representative markers of airway remodeling. Overall, the above data indicated that ICA did contribute to anti-remodeling, but the definite functional mechanism of ICA remains unclear and needs to be further clarified.

Despite the myriad of studies in this field, it remains elusive how ICA is resistant to airway remodeling at the molecular level and which downstream molecules and signaling pathways are involved. The main alterations in remodeling include the destruction of airway epithelial integrity, deposition of the extracellular matrix, neovascularization, vascular remodeling, mucinous gland hyperplasia, and especially ASMC hyperplasia and hypertrophy [2].

The potential role of ASMC has been comprehensively reviewed by other researchers [54, 55]. It is generally believed that the total amount of ASMC is abnormally increased in asthma and associated with the duration and severity of asthma [56]. Theoretically, augment of airway smooth muscle mass can be achieved by cell proliferation, cell hypertrophy, or a combination of both [57]. In this study, the results of ELISA demonstrated that ICA inhibited the release of IL-13 and ET-1 in BALF and serum. IL-13 is a Th2 type cytokine produced by activated T cells, basophils or mast cells. IL-13 is an important mediator of allergen-induced AHR and regulates ASMC directly and alone, rather than relying on inflammation [58]. Also, various investigators have detailed that IL-13 assumes a key job in the effects of airway remodeling induced in ASMC, fibroblasts, endothelium and epithelium. ET-1 is a well-known mitogen and contractile agonist for ASMC [46], increased levels of ET-1 in the bronchoalveolar fluid have been associated with AHR and severity of asthma [59]. The study of Lan, B.etc. indicates that mechanical compression of bronchial epithelial cells contributes to proliferation and basal contraction of ASMC, and that augmented proliferation and contraction depend on epithelial cell-derived ET-1 [46]. Since IL-13 and ET-1 are regulated by ICA and strongly associated with ASMC proliferation in airway remodeling, whether ICA has an effect on ASMC proliferation has not yet been clarified. To this end, flow cytometry was used to examine the effect of ICA on ASMC, and the data showed that ICA can inhibit the proliferation of ASMC. Whereas, the in-depth mechanism by which ICA inhibits cell proliferation has remained an open question.

Cell proliferation and differentiation are the results of a combination of factors. Among these factors, the signal transduction pathways are extremely complex. Signal transduction pathways that have been confirmed to have regulatory effects are MAPK/Erk signaling pathway, JAK-STAT signaling pathway, PI3K-Akt signaling pathway, PLCβγ/PKCβ/PKD signaling pathway, etc. [60]. It is confirmed that the MAPK/Erk signaling pathway is involved in a variety of cellular activities, such as the proliferation, migration, differentiation, and apoptosis [61]. In the present study, ICA reduced mRNA levels of Erk1/2 and p21ras in ASMC in a dose-dependent manner. Since Erk1/2 and p21ras are key members of MAPK signaling pathway, our results may suggest that targeting MAPK/Erk signaling pathway may be a mechanism by which ICA inhibits ASMC proliferation.

## Conclusion

In summary, this study demonstrated that ICA inhibited ASMC proliferation in OVA-induced airway remodeling through inhibiting the release of TGF-β1, VEGF, IL-13, ET-1 and the activity of MAPK/Erk signaling pathway. The mechanisms revealed in this study are of significance in the treatment of diseases associated with airway remodeling, such as asthma.

## Supplementary information

**Additional file 1. **Completed **“**The ARRIVE Guidelines Checklist**”** for reporting animal data in this manuscript.)

## Data Availability

The data analyzed during the current study are available from the corresponding author on reasonable request.

## Abbreviations

AHR: Airway hyperresponsiveness
AIOD: Average integral optical density
ASMC: Airway smooth muscle cell
BALF: Bronchoalveolar lavage fluid
DXM: Dexamethasone
ET-1: Endothelin-1
H&E: Hematoxylin and eosin
ICA: Icariin
IHC: Immunohistochemistry
IL-13: Interleukin 13
MAPK: Mitogen-activated protein kinase
N: Amount of ASMC nucleus
OD: Optical density
OVA: Ovalbumin
Pbm: Basement membrane perimeter
Penh: Enhanced pause
TGF-β1: Transforming growth factor beta 1
VEGF: Vascular endothelial growth factor
Wai: Inner airway area
Wam: Smooth muscle area
